# Long-latency Responses to a Mechanical Perturbation of the Index Finger Have a Spinal Component

**DOI:** 10.1523/JNEUROSCI.1901-19.2020

**Published:** 2020-05-13

**Authors:** Demetris S. Soteropoulos, Stuart N. Baker

**Affiliations:** Institute of Neuroscience, Newcastle University, Newcastle, NE2 4HH, United Kingdom

**Keywords:** corticospinal, long-latency response, motor cortex, perturbation, spinal cord

## Abstract

In an uncertain external environment, the motor system may need to respond rapidly to an unexpected stimulus. Limb displacement causes muscle stretch; the corrective response has multiple activity bursts, which are suggested to originate from different parts of the neuraxis. The earliest response is so fast, it can only be produced by spinal circuits; this is followed by slower components thought to arise from primary motor cortex (M1) and other supraspinal areas. Spinal cord (SC) contributions to the slower components are rarely considered. To address this, we recorded neural activity in M1 and the cervical SC during a visuomotor tracking task, in which 2 female macaque monkeys moved their index finger against a resisting motor to track an on-screen target. Following the behavioral trial, an increase in motor torque rapidly returned the finger to its starting position (lever velocity >200°/s). Many cells responded to this passive mechanical perturbation (M1: 148 of 211 cells, 70%; SC: 67 of 119 cells, 56%). The neural onset latency was faster for SC compared with M1 cells (21.7 ± 11.2 ms vs 25.5 ± 10.7 ms, respectively, mean ± SD). Using spike-triggered averaging, some cells in both regions were identified as likely premotor cells, with monosynaptic connections to motoneurons. Response latencies for these cells were compatible with a contribution to the muscle responses following the perturbation. Comparable fractions of responding neurons in both areas were active up to 100 ms after the perturbation, suggesting that both SC circuits and supraspinal centers could contribute to later response components.

**SIGNIFICANCE STATEMENT** Following a limb perturbation, multiple reflexes help to restore limb position. Given conduction delays, the earliest part of these reflexes can only arise from spinal circuits. By contrast, long-latency reflex components are typically assumed to originate from supraspinal centers. We recorded from both spinal and motor cortical cells in monkeys responding to index finger perturbations. Many spinal interneurons, including those identified as projecting to motoneurons, responded to the perturbation; the timing of responses was compatible with a contribution to both short- and long-latency reflexes. We conclude that spinal circuits also contribute to long-latency reflexes in distal and forearm muscles, alongside supraspinal regions, such as the motor cortex and brainstem.

## Abstract (precis)

In an uncertain external environment, the motor system may need to respond rapidly to an unexpected stimulus. Limb displacement causes muscle stretch; the corrective response has multiple activity bursts, which are suggested to originate from different parts of the neuraxis. The earliest response is so fast, it can only be produced by spinal circuits; this is followed by slower components thought to arise from primary motor cortex (M1) and other supraspinal areas.

## Introduction

The spinal cord (SC) is often considered to be the epicenter of limb reflexes, as it contains the necessary neural circuits for mediating a varied repertoire of very fast responses to an external stimulus. This is exemplified by the classical stretch reflex, whereby a mechanical stimulus causes the rapid lengthening of a muscle, which in turn causes the same muscle to contract with an onset too fast to be mediated through any system except the SC.

Muscle responds to stretch with multiple bursts of activity. The earliest component, referred to as the short-latency response (SLR), is mediated through fast Group Ia spindle afferents with conduction velocities at ∼85 m/s. Primary spindle afferentsare particularly sensitive to rapid changes in muscle length (Matthews, 1972), are reliably activated during a mechanicalperturbation, and have direct and potent monosynaptic connections onto motoneurons (Landgren et al., 1962; Mendell and Henneman, 1968; Jack et al., 1971). After the SLR contributions from the SC are typically considered to be over, any later muscle activity, usually termed the long-latency response (LLR, also used as such in our paper), is thought to be mediated through supraspinal systems.

One such supraspinal system is the primary motor cortex (M1). Neurons in M1 respond to peripheral stimulation (Lemon and Porter, 1976a,b) with a short delay, and in turn there are numerous projections from M1 back down to the SC. In primates, some M1 cells directly contact motoneurons (Maier et al., 1993; Lemon et al., 1998). These cortico-motoneuronal (CM) cells respond to mechanical perturbations in distal joints at delays compatible with a contribution to the LLR in monkey forearm muscles (Cheney and Fetz, 1984). The transcortical component of the LLR probably partly explains its sensitivity to varying task demands (Omrani et al., 2014; Pruszynski, 2014; Pruszynski et al., 2014). Other possible contributors include subcortical areas with their own descending pathways to the SC, such as the brainstem reticular formation (RF) and red nucleus (Soteropoulos et al., 2012; Herter et al., 2015).

**Figure 1.**
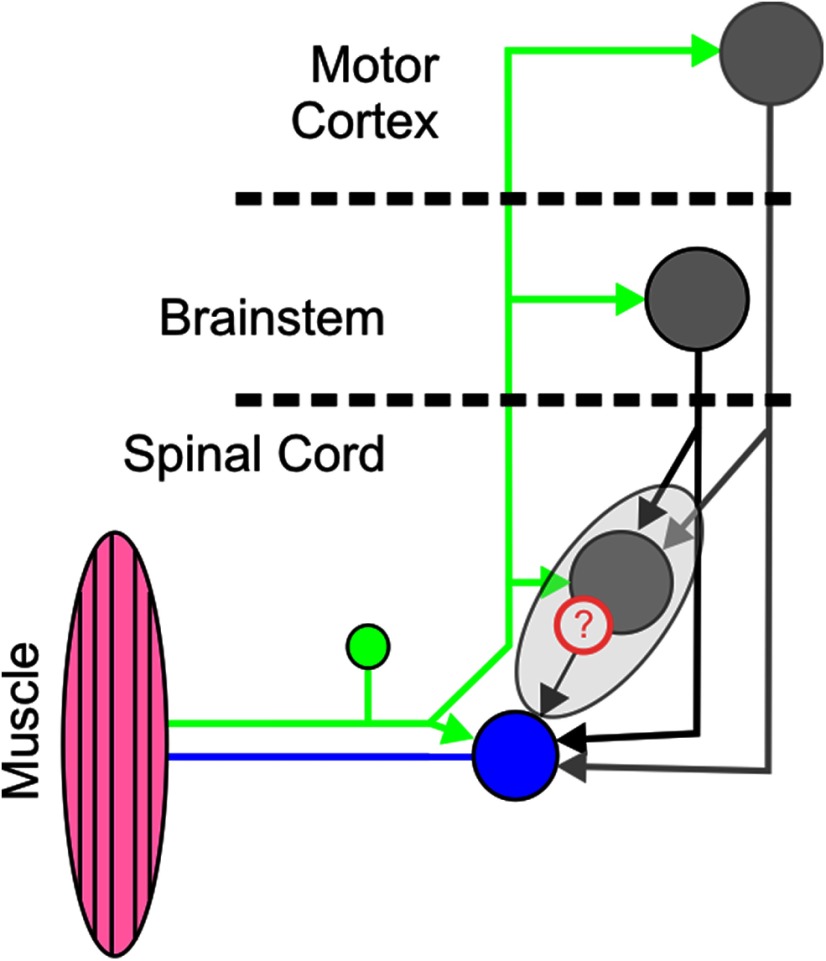
Schematic of possible circuits to mediate LLRs to perturbation. Blue represents a spinal motoneuron. Gray represents three different premotoneuronal sources (SC, brainstem, and cortex). The question mark is to highlight the lack of knowledge regarding the potential role of spinal interneurons to contribute to activity in muscles following a perturbation despite it being well established that many spinal neurons are in receipt of many afferent and descending inputs.

Given the involvement of spinal neuronal circuits in complex motor actions, spinal contributions to components later than the monosynaptic stretch reflex would be to be expected (Fig. 1). Indeed, muscle responses at LLR delays survive interruption of the transcortical route in both monkeys and cats (Ghez and Shinoda, 1978; Tracey et al., 1980; Miller and Brooks, 1981). Similar evidence also exists in humans with SC injury (Roby-Brami and Bussel, 1987). Many spinal interneurons in the intermediate layers of the SC receive sensory inputs from several afferent classes (Brown and Fyffe, 1978, 1979; Fyffe, 1979; Lundberg, 1979b; Jankowska et al., 1981; Jankowska and McCrea, 1983; Jankowska and Edgley, 2010), and many in turn also provide a potent source of inputs to motoneurons (Fetz et al., 1996; Takei and Seki, 2010, 2013; Williams et al., 2010).

Disentangling the specific contribution of afferents inputs through spinal interneurons is hard to do in humans, particularly for the upper limb (but see Pierrot-Deseilligny and Burke, 2012). Manipulations, such as peripheral cooling of the arm (Matthews, 1989) and pharmacological interventions using tizanidine (Marque et al., 2005; Lourenco et al., 2006; Meskers et al., 2010), have shown a potential Group II involvement in the LLR, but results have not been consistent (see Kurtzer et al., 2018). Other studies provide support for a cutaneous afferent contribution to the LLR (Corden et al., 2000).

In order to assess the importance of spinal circuits to the LLR, it is necessary to show that spinal circuits are activated following a perturbation during behavior, and to relate the onset and duration of the SC responses to that of the muscles. In the reported experiments, we recorded the responses of SC cells to a mechanical perturbation of the index finger in the awake behaving monkey, and compared their firing with that of M1 cells. A substantial fraction of SC neurons responded to index finger perturbations; the timing of discharge modulation was consistent with a contribution to both early and late components of the muscle response.

## Materials and Methods

All animal procedures were performed under UK Home Office regulations in accordance with the Animals (Scientific Procedures) Act (1986) and were approved by the relevant Local Research Ethics Committee. Experiments were conducted on 2 adult female purposed-bred macaque monkeys (*Macaca mulatta*). Animals were pair-housed and had *ad libitum* access to water at all times. Food access was restricted during training and recordings but was *ad libitum* during the weekend. If the number of rewards taken during recordings fell below a threshold level for 2 consecutive days, animals were given *ad libitum* access to food on the second day.

### 

#### Behavioral task

The task has been described previously (Williams et al., 2009, 2010; Soteropoulos et al., 2012). Briefly, animals were trained to perform a slow index finger movement using visual feedback. The hand, and digits 1 and 3-5, rested within a padded pocket, which constrained movements in all directions while the index finger was used to perform flexion/extension movements through movements across the metacarpophalangeal joint. The index finger pressed on a lever attached to the shaft of a torque motor and optical encoder, mounted approximately coaxially with the metacarpophalangeal joint. The target appeared at a stationary position (HOLD 1, 1 s), moved with a constant velocity of 12°s^−1^ (movement period, RAMP) for 1 s, and then remained stationary for a further 1 s (HOLD 2). Movements were either in the extension or flexion direction, chosen randomly; the HOLD 1 and HOLD 2 displacements required flexion by 12° or 24° from the neutral position. The lever was attached to a motor, which simulated a spring load (torque for initial lever movement, 26.4 mN m; spring constant, 1.8 mN m deg^−1^). Force on the lever was always in a direction to oppose finger flexion. Deviations from the target (typically > 1.4°) resulted in an error signal, and the trial was terminated with no reward. At the end of a correct trial, or after an error during the trial, the lever was rapidly (peak velocity typically in excess of 100°/s) returned to the start position by increasing the torque to the motor. If the trial was successful, the monkey was given a food/liquid reward. The working arm was gently supported in a sleeve to prevent proximal movements, while the contralateral arm was not restrained.

#### Surgery and implants

Following behavioral training, monkeys were implanted with a stainless-steel headpiece to allow atraumatic head fixation. Recording chambers were over the M1 (chamber center 12 mm anterior to the interaural line and 18 mm lateral to midline) to allow single-unit recordings from this area. Animals were prepared for EMG recording by implantation with up to 10 epimysial patch electrodes, sutured to hand and forearm muscles (see below for list of muscles). Wires from these electrodes led subcutaneously to a connector mounted on the animal's back. Following recordings from M1 and the RF (reticular data are not part of this paper), a spinal chamber was implanted over the cervical SC, involving fusion of vertebrae from C4 to T2 (Williams et al., 2010).

To allow antidromic identification of corticospinal neurons (pyramidal tract neurons [PTNs]), stainless-steel stimulating electrodes insulated with parylene (MS501G, Microprobe) were implanted in the medullary pyramidal tract (PT) ipsilateral to the recorded M1, using a double-angle stereotaxic technique (Soteropoulos and Baker, 2006), with initial targets A0 ML 0.7 DV −6 relative to interaural line. During electrode placement, antidromic volleys were recorded from epidural electrodes placed over M1.

All procedures were performed using aseptic technique under general anesthesia comprising 3%-5% inhaled sevoflurane in 100% O_2_, supplemented with a continuous intravenous infusion of alfentanil (25 μg kg^−1 h−1^). Postoperative care included broad-spectrum antibiotic cover: coamoxyclav 140/35 (Synulox); clavulanic acid 1.75 mg kg^−1^, amoxycillin 7 mg kg^−1^ (Pfizer); cefalexin (Ceporex, 10 mg kg^−1^, Schering‐Plough Animal Health); amoxycillin (Clamoxyl LA), 15 mg kg^−1^ (Pfizer), analgesics (buprenorphine, Vetergesic, 10 mg kg^−1^, Reckitt and Colman); and carprofen (Rimadyl, 5 mg kg^−1^, Pfizer).

#### Behavioral, neural, and muscle recordings

During performance of the task, the angular position of the lever was recorded (500 Hz sampling rate) concurrently with the other neurophysiological signals described below. Various task and behavioral events (e.g., the trial, hold, and move onset) were also captured.

Extracellular activity was recorded from multiple neurons from the contralateral M1 and the ipsilateral SC, relative to the hand performing the task. From M1, recordings were made using a 16-channel Eckhorn drive system, while from the SC a 5-channel mini matrix drive was used, loaded with tetrodes (all from Thomas Recording).

Cortical recordings targeted the hand representation of M1. Intracortical microstimulation (trains of 13 biphasic pulses, 0.2 ms per phase, 3 ms interspike interval, intertrain interval of ∼1s, stimulation intensity <40 μA) was typically conducted at the start and end of each penetration to verify this.

Spinal recordings targeted segments C7 to T1, ipsilateral to the moving finger. Estimation of the depth of the electrodes relative to the surface of the dura for spinal recordings is prone to errors due to the progressive thickening of the dura and overlying tissues over successive days. To accommodate for this, for each spinal penetration, the depth of the first cellular activity was noted which would likely correspond to the dorsal horn. The depth of ensuing cell recordings for that session was expressed relative to this. As with M1 recordings, microstimulation (intensity up to 50 μA) was performed at the end of each recording session; and by observing evoked movements and/or muscle activity, we could verify that we were recording from the appropriate cervical segments and regions.

Waveform signals were filtered and stored (0.3–10 kHz bandpass filter, gain 2000–10,000 sampling rate 25 kHz) for offline analysis in conjunction with the behavioral task signals. Local field potentials were also recorded from the same electrodes (3–100 Hz bandpass filtered, 1000–5000 gain, 500 Hz sampling rate).

EMG recordings were available from subcutaneous patch electrodes implanted over the following muscles: flexor digitorum superficialis (FDS), flexor digitorum profundus (FDP), flexor carpi radialis (FCR), flexor carpi ulnaris (FCU), extensor carpi ulnaris (ECU), extensor digitorum communis (EDC), extensor carpi radialis (ECR), abductor pollicis longus (AbPL), first dorsal interosseus (1DI), and abductor pollicis brevis (AbPB, Monkey D only). As AbPB and AbPL had very weak and infrequent responses to the perturbation and they did not control the digit being perturbed, their responses will not be considered further in the paper. EMG signals were sampled at 5 kHz (gain 500–2000 K, 30 Hz to 2 kHz bandpass). Before any analysis, raw EMG signals had any DC offset removed and were then full wave rectified.

Neurons in M1 were identified as corticospinal if they responded to single-pulse stimulation of the PT (stimulus intensity < 400 μA, biphasic pulse, 0.2 ms duration per phase, repetition rate ∼1Hz) with a constant (<0.1 ms jitter) antidromic latency and if they showed a constant collision interval (as in Lemon, 1984).

#### Testing of peripheral inputs

For the spinal recordings, some of the neural responses to peripheral stimulation were tested, either before or after the recording session. This was conducted by manipulating the surface of the skin, muscles, and joints of the fingers, wrist, and arm to establish the receptive field of the unit. In many cases, it was possible to distinguish between cutaneous and deep modalities, and the presence or absence of a receptive field could be readily confirmed.

#### Analysis

The response of neurons to a particular event or stimulus was analyzed by compiling perievent time histograms (PETHs), whereby neural activity was aligned to the event of interest, binned (nonoverlapping bins, width = 1 ms, unless otherwise stated), and averaged across trials. This was then smoothed by convolving with a Gaussian kernel (unit area, width parameter 2 ms), which then allowed measurement of response latency and amplitude relative to the event of interest.

Lever velocity and acceleration were estimated by differentiating the lever position signal once and twice, respectively. The time of peak velocity was used initially to align neural and muscle activity across trials. The estimated mean onset latency of the perturbation for a given recording session was then taken as the time point at which the lever acceleration exceeded 2 SDs from baseline.

The activity of muscles and many cells was not always stationary around the time of the perturbation, and this could make estimating onset latency and response amplitude problematic. To compensate for this, a regression line was fitted to a 100 ms epoch before the perturbation. This line was then extrapolated to 100 ms after perturbation onset and subtracted from the response.

The onset latency of EMG and cell responses to the perturbation was then estimated by taking the first instance within the first 75 ms after stimulus where all values were larger or smaller than the adjusted baseline for at least 5 ms. The mean value within this 5 ms epoch was then compared (*t* test, *p* < 0.005) with the distribution of mean values of 200 randomly selected epochs of the same width from the prestimulus region.

Cells in both M1 and SC with direct connections onto motoneurons (CM cells; or spinal premotor cells [PM]) were identified through spike-triggered averaging of EMG for each muscle recorded from. Significant postspike effects were detected as described by Williams et al. (2010). To avoid spurious effects in these averages caused by the fact that both cells and muscles responded to the perturbation, spikes that occurred 0–100 ms after the perturbation were excluded when compiling the averages.

#### Response ratio between areas

It was of interest to compare the fraction of cells that were active at various delays after perturbation. For each cell, after the subtraction of baseline activity from the PETH (as described above), the SD was estimated from the 100 ms preperturbation epoch, and any bins that were larger or smaller than the 95% confidence limits were considered as significantly modulated. This was then used to create an estimate of what fraction across a population of cells was active at any given time point after perturbation.

It was also of interest to examine how the ratio of responsive cells active between the two recorded areas evolved over time, as that could give an indication of whether one group of cells was more or less involved than the other at a given epoch. This was simply estimated by the following:

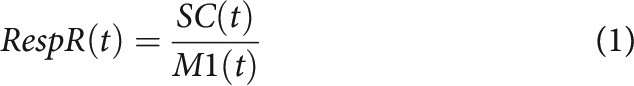


Whereby SC(t) is the fraction of responsive cells in the SC at time t that are significantly different from baseline; M1(t) is the same but for M1. Values >1 indicate that a higher fraction of spinal cells were active compared with M1 at a given time, and the inverse is true for values <1. In order to detect whether the *RespR* was significantly different from 1, a shuffling approach was taken whereby the identity of cells (SC vs M1) was randomly shuffled 500 times, before the same calculation was conducted. If the *RespR* value of the real data were outside the 95% confidence limits of the shuffled data, then the ratio was considered significantly different from 1.

#### Modulation of cell activity with the task

The activity of M1 cells during the task has been discussed in previous work (Williams et al., 2009; Soteropoulos et al., 2012). For the present study, it was of interest to examine whether cells responded differentially for flexion versus extension trials, as that would indicate activity specific to task performance rather than a general response to movement (e.g., postural stabilization). To do this, PETHs were generated aligned to the end of the successful flexion and extension trials separately, and smoothed with a Gaussian kernel (width parameter 2 ms). From these PETHS, we calculated the directionality index (DI) as follows:

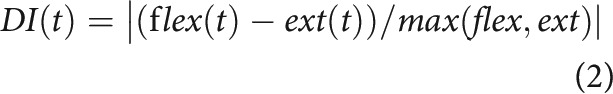


Whereby the DI at time *t* was the absolute difference between the firing rate during flexion (*flex*) and extension (*ext*) trials at time *t*, divided by the maximal rate seen during either trial. A DI value of zero indicates an identical rate during extension and flexion trials; values close to 1 indicate a substantial difference between the two trial types. For each cell, the mean values of the DI during the three task phases (first hold, ramp, second hold) were averaged to generate a mean DI for that neuron. To detect whether this was significantly different from zero, 200 shuffled PETHs for flexion and extension trials were generated where the identity of the trial was randomized, and the mean DI was recalculated for each shuffle. If the DI value of the real data were >10 of the DI values from shuffled data, then the DI was considered significantly different from zero (*p* < 0.05).

#### Estimates of peripheral and central delays

In order to estimate the potential contribution of neural activity in M1 and SC to responses in muscles, an estimate of the peripheral conduction delays was required. To obtain this, we estimated the latency of the muscle responses to PT stimulation, which was typically conducted during the M1 recording sessions to test for PTN cells. From each onset latency, we subtracted 1.7 ms, comprising the conduction delay from the PT electrode to the SC (typically ∼0.6 ms), a 1 ms synaptic delay from corticospinal axons to motoneurons, and an additional 0.1 ms utilization time to allow for the PT stimulus to activate PT axons. This latency is an estimate of the fastest efferent delay from SC to muscle. To estimate the afferent delay from muscle to cord, we needed to scale the calculated efferent delay by the ratio of the conduction velocities of Group Ia afferents (∼85m/s) (Cheney and Preston, 1976) and motor efferents (∼75 m/s) (Eccles et al., 1968). The sum of the efferent and afferent delays plus 1 ms for synaptic transmission allowed us to estimate the fastest possible latency for the monosynaptic stretch reflex for each muscle, excluding the mechanical delay for spindle activation.

## Results

A total of 211 neurons were recorded from M1 (Monkey D:102, Monkey R:109), from 66 penetrations (39 in Monkey D, 27 in Monkey R). Sixty M1 cells were identified as PTNs (30 in Monkey D, 30 in Monkey R) and an additional 19 as CM cells (6 in Monkey D, 13 in Monkey R). Unidentified (UIDs) neurons were recorded from the same penetrations and locations as PTN/CM cells and were thus likely also to be layer V pyramidal cells. From the SC, 42 penetrations (18 in Monkey D, 24 in Monkey R) yielded 119 neurons (24 in Monkey D and 95 in Monkey R), with 26 of those identified as PM cells (3 in Monkey D, 23 in Monkey R). The yield of CM/PM cells is comparable to yields from previous studies (e.g., Takei and Seki, 2010) as identification of such cells necessarily only happens after the data are collected.

### Muscle responses to mechanical perturbation

**Figure 2.**
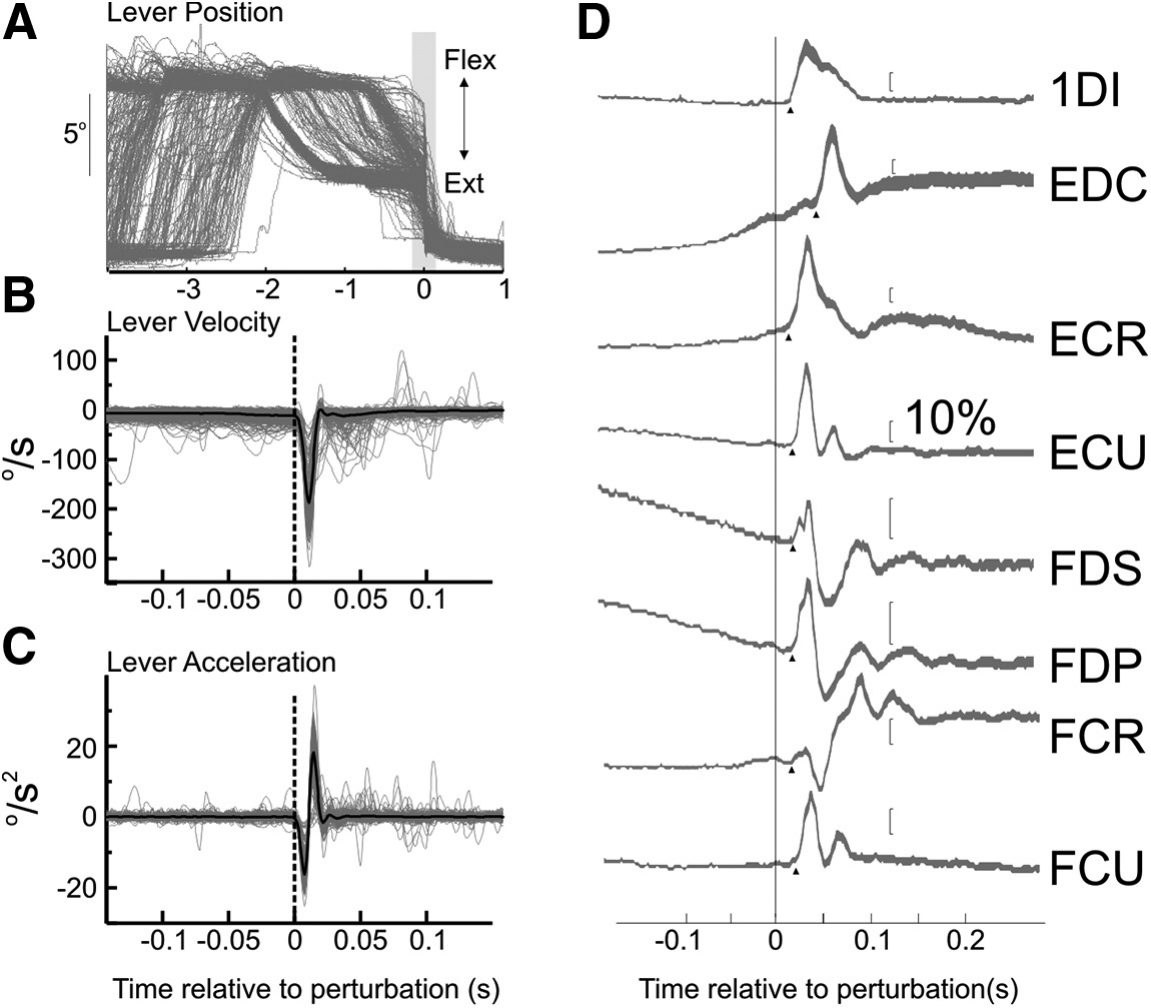
Task signals during perturbation and example EMG responses. ***A***, Overlain lever position traces aligned relative to the perturbation onset. ***B***, Velocity traces of expanded epoch around the perturbation. ***C***, Lever acceleration. ***D***, Mean rectified EMG signals recorded from the same session. Calibration bars for each muscle correspond to 10% of the mean EMG preperturbation epoch (100 ms).

The rapid return of the lever produced peak lever velocities often in excess of 200°/s. For >88% of recording sessions in both M1 and SC, the mean peak velocity across all trials was >200°/s (range of mean peak values: 115–442°/s). Figure 2*A* shows the lever position signals for a typical recording session in gray, aligned to peak velocity. The gray shaded area is expanded in Figure 2*B*, which now shows the velocity traces of the lever; for this session, many trials had a peak velocity in excess of 200°/s. Figure 2*C* shows the lever acceleration. The perturbation was sufficient to produce a robust response in this session from all recorded muscles (Fig. 2*D*).

**Figure 3.**
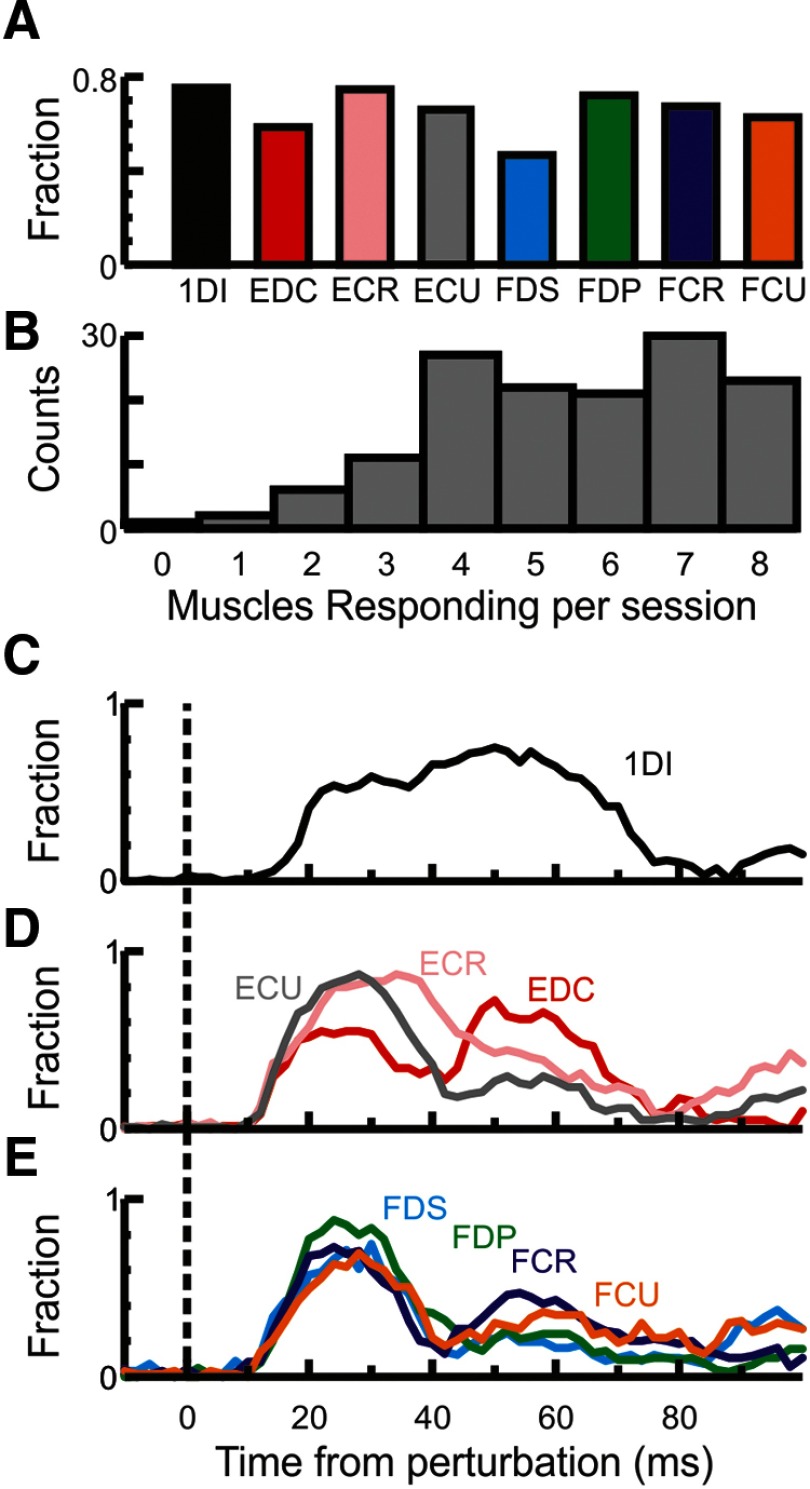
Response incidence in recorded muscles. ***A***, The incidence of responses for each muscle across all recording sessions. ***B***, Total number of muscles showing a response per recorded session. ***C***, Temporal profile of the response for 1DI across sessions, where each time point shows the fraction of all sessions, which had a value larger than 2× SD of the background epoch. ***D***, Same as in ***C***, but for the muscles in the extensor compartment in the forearm. ***E***, Same as in ***C***, but for the muscles in the flexor compartment in the forearm. For all muscles recorded, the response to the perturbation continued beyond 70 ms after perturbation.

The response incidence for each muscle is shown in Figure 3*A*; Figure 3*B* illustrates the number of muscles responding in a session. Almost all (98.6%) sessions showed a response in at least one recorded muscle, while most sessions (80%) showed a response in at least two of the intrinsic hand and forearm flexor muscles (1DI, FDS, FCU, FCR, or FDP). Figure 3*C–E* shows the temporal profile of the response for the different muscles across sessions; at each time point is plotted the fraction of all sessions with EMG larger than 2× SD of the background epoch. For all muscles recorded, the response to the perturbation continued for >70 ms after the perturbation.

**Figure 4.**
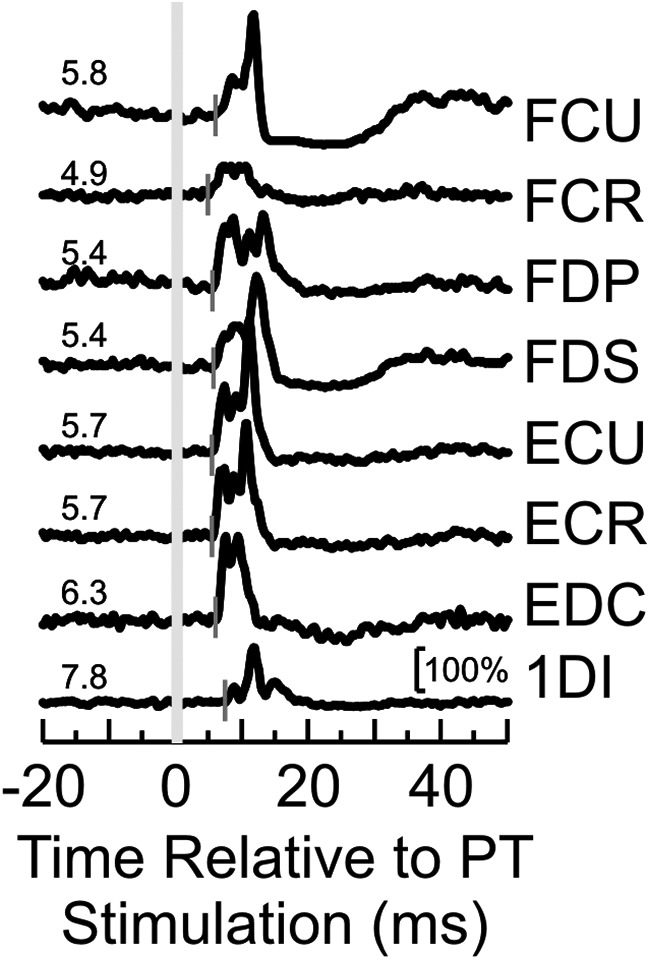
Muscle responses to PT stimulation. Example rectified EMG responses from recorded muscles following single-shock PT stimulation at 300 μA (long gray line). Numbers next to each response indicate the onset latency of the response in ms (short gray lines).

**Figure 5.**
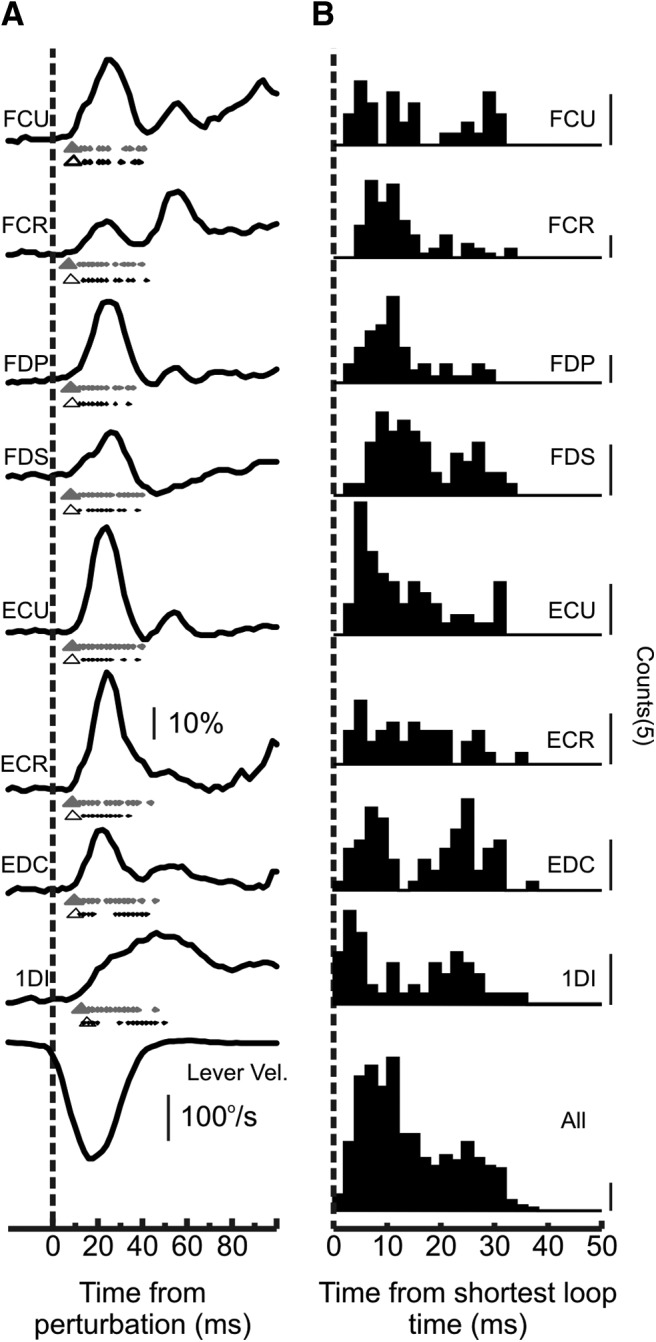
Estimated response latencies of muscles. ***A***, Mean muscle response aligned to the onset of the perturbation. EMG responses scaled to the preperturbation epoch (100 ms). Bottom, Velocity trace average. Under each muscle trace is the estimated peripheral loop time for each muscle (triangle: black for Monkey D; gray for Monkey R); the dots represent response latencies for the given muscle measured from individual recording sessions. ***B***, Histogram of the response latency for each muscle aligned relative to the peripheral loop time for the given muscle. Bottom, A similar histogram combined across all muscles.

In order to determine the possible contributions of neural activity to muscle responses, we needed an estimate of peripheral conduction time. Figure 4 shows exemplar EMG responses to PT stimulation that were used to estimate the efferent delay from motoneuron to muscle (see Materials and Methods). Figure 5*A* shows the mean response for different muscles, aligned to the onset of the perturbation. EMG responses are scaled as a percentage of the 100 ms period before the perturbation. Under each muscle trace is the estimated peripheral loop time for each muscle (triangle, black for Monkey D and gray for Monkey R). Dots show response latencies for that muscle, measured from single sessions in each animal. The peripheral delay estimates were typically consistent to within 1.3 ms between the 2 monkeys. The exception to this was the 1DI muscle, for which Monkey R had an estimated loop time of 12.4 ms compared with 15.5 ms for Monkey D. Monkey D was the larger of the two (8 vs 5.2 kg); this difference may in part represent the extra conduction delay associated with longer arms. An additional contribution to the difference between animals could come from differences in the implantation location of the recording wires relative to the motor point within the muscle. For forearm muscles, peripheral loop times were 7–10.3 ms. Figure 5*B* shows a histogram of the response latency after the perturbation for each muscle, aligned relative to the respective peripheral loop time. The bottom histogram shows a combined histogram for all muscle responses.

These analyses show that the various muscles controlling the index finger were robustly activated from the perturbation with onset delays consistent with activation of fast afferents.

### Neural responses to mechanical perturbation

**Figure 6.**
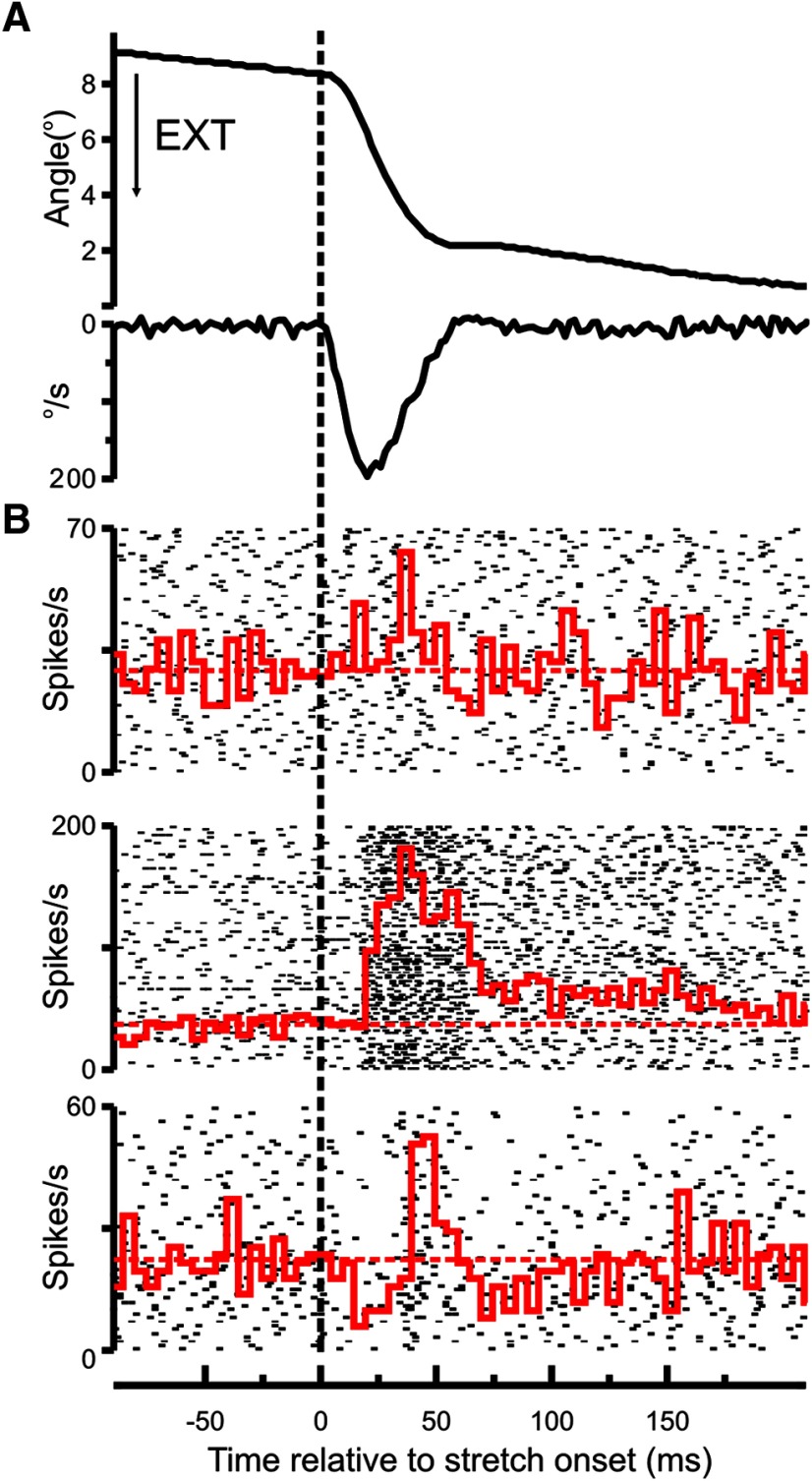
Example responses in spinal interneurons. ***A***, Average position (top) and velocity (bottom) traces for a single recording session. Arrow indicates the direction of movement that causes a finger extension. ***B***, The PETH for three cells recorded from the SC (in red) overlain on the corresponding raster plots. Horizontal dotted line indicates the preperturbation firing rate.

In both M1 and SC, a substantial (>50%) fraction of neurons showed a significant response to the perturbation. Figure 6 shows the PETH and raster plots of three SC neurons, with their activity aligned to the onset of the perturbation. Across all cells, a minimum of 18 perturbations were used to generate the PETHs (mean 481, range 18-1102).

**Figure 7.**
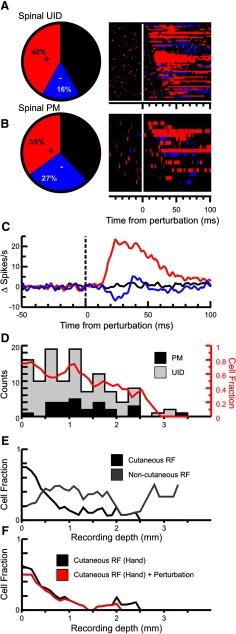
Response incidence in the SC. ***A***, Pie chart showing the fraction of spinal UID cells responding to the perturbation with an increase in rate (red), a decrease (blue), or no response (black). The raster to the right of the pie chart shows the bins for each neuron that were larger (red) or smaller (blue) than twice the SD of the preperturbation epoch (100 ms). Cells have been sorted by response latency. ***B***, Same as in ***A***, but for identified spinal premotor (PM) cells. ***C***, Mean PETH across all spinal cells. Red represents the mean response across all cells with a positive response. Blue represents those with a negative response. Black represents the mean for those with no significant response. Baseline activity has been subtracted from each cell before averaging. ***D***, Depth distribution of all recorded spinal cells. Gray represents the UIDs. Black represents the PM cells. Red trace represents a sliding average of the fraction of cells at a given depth that responded to the perturbation. ***E***, Depth distribution of cells responding to cutaneous stimulation (black) and to deep stimulation (gray). ***F***, Depth distribution of cells responding to cutaneous stimulation in the hand (black line), and those that also responded to the finger perturbation (red line).

The frequency of responses to the perturbation is shown as pie charts for UID cells in the SC in Figure 7*A*. The frequencies for premotor interneurons identified through spike-triggered averaging of the activity of eight muscles are shown Figure 7*B*. Most of the spinal PM cells (23 of 26) showed postspike facilitation. More than half of all spinal neurons (56%) showed a significant response to the perturbation; and of those, 67% showed an increase in firing rate as the earliest component of their response (red pie chart sections). To the right of the pie charts are rasters representing the responses of individual cells. Bins are colored red if > 2 × SD of the baseline, and blue if <−2 × SD. Figure 7*C* shows the mean responses of cells to the perturbation, with the baseline subtracted as discussed in Materials and Methods. Red, blue, and black lines indicate averages for cells with a significant increase, decrease, or no change in firing after to the perturbation (positive, negative, or unmodulated cells). There was no significant difference (one-way ANOVA, *F* = 0.44, *p* = 0.64) in the baseline activity between positive, negative, and unmodulated cells (mean baseline values of 24.6, 28.9, and 23.6 Hz, respectively). Positive cells had a significantly (*p* < 0.001, unpaired *t* test) larger response amplitude (mean 21 Hz above baseline) compared with negative cells (mean 7.1 Hz below baseline). However, as response amplitude is expressed relative to baseline, then there is a flooring bias for negative cells (firing rate cannot go lower than zero).

Figure 7*D* is a histogram of the depth that cells were recorded from, with gray showing all cells and black identified PM cells. The line in red corresponds to the fraction of cells at a given depth that responded to the perturbation. There was a significant correlation (Pearson's correlation, *R*^2^ = 0.76, *p* < 0.0003) between incidence of response and depth of recording. Although we cannot definitely identify the laminar location of our neural recordings, cells recorded from the more superficial depths had a much higher incidence of response compared with deeper recordings, which is consistent with the known termination pattern of sensory afferents in the SC (Molander and Grant, 1985; Woolf and Fitzgerald, 1986; Edgley and Jankowska, 1988; Riddell and Hadian, 2000; Fisher et al., 2020).

For some of the spinal cells (*n* = 95), we were able to test responses to peripheral stimulation and found that a majority (73%) was responsive. Of the PM cells that were tested (*n* = 18), the fraction responding to peripheral stimulation was 61% (*n* = 11). Figure 7*E* shows the depth distribution of cells with cutaneous (black) and deep (gray) receptive fields. At depths that would correspond to superficial layers of the SC (<0.5 mm), most (85%) of the tested SC cells responded to peripheral stimulation and of those, most responded to cutaneous stimulation (69%). Figure 7*F* is focused on cells responding to cutaneous inputs from the hand (black line), and a similar pattern as Figure 7*E* is seen. Of the 25 cells with responses to cutaneous stimulation in the hand, most (*n* = 21, 84%) also responded to the finger perturbation (red line).

**Figure 8.**
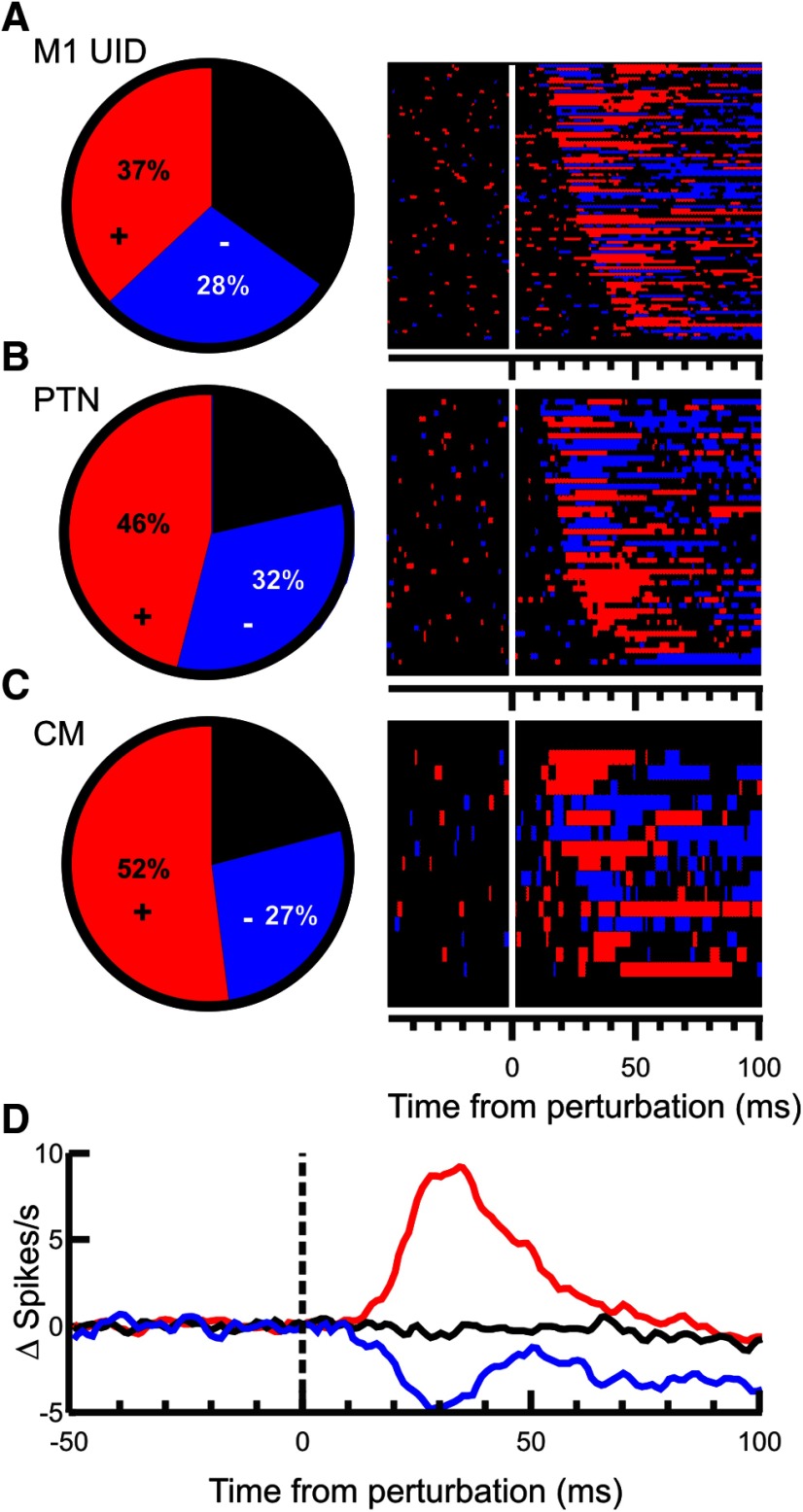
Response incidence in the M1. ***A***, Pie chart showing the fraction of M1 UID cells responding to the perturbation with an increase in rate (red) or a decrease (blue) or no response (black). The raster to the right of the pie chart shows the bins for each neuron that were larger (red) or smaller (blue) than twice the SD of the preperturbation epoch (100 ms). Cells have been sorted by response latency. ***B***, Same as in ***A***, but for identified PTNs. ***C***, Same as in ***A***, but for identified CM cells. ***D***, Mean PETH across all M1 cells. Red represents the mean response across all cells with a positive response. Blue represents those with a negative response. Black represents the mean for those with no significant response. Baseline activity has been subtracted from each cell before averaging.

Figure 8 is a comparable figure but for the M1 cells. More than two-thirds of all M1 cells (70%) showed a significant response to the perturbation; and of those, 59% showed an increase in firing rate as the earliest component of their response. Figure 8*A* shows a pie chart of the fraction of M1 UID cells responding to the perturbation, and a raster indicating the response timing. Similar plots for PTNs and CM cells are shown in Figure 8*B*, *C*. Figure 8*D* shows the mean responses of cells to the perturbation. Unlike for spinal cells, there was a significant difference between the baseline firing rate of positive (mean 14.9 Hz), negative (19.6 Hz), and unmodulated (10.8 Hz) cells (one-way ANOVA, *F* = 8.59, *p* = 0.0003). As with spinal cells, positive cells had a significantly (*p* < 0.001, unpaired *t* test) larger response amplitude (8.8 Hz) compared with negative cells (5.1 Hz), although the same flooring caveat applies as described previously for SC cells.

**Figure 9.**
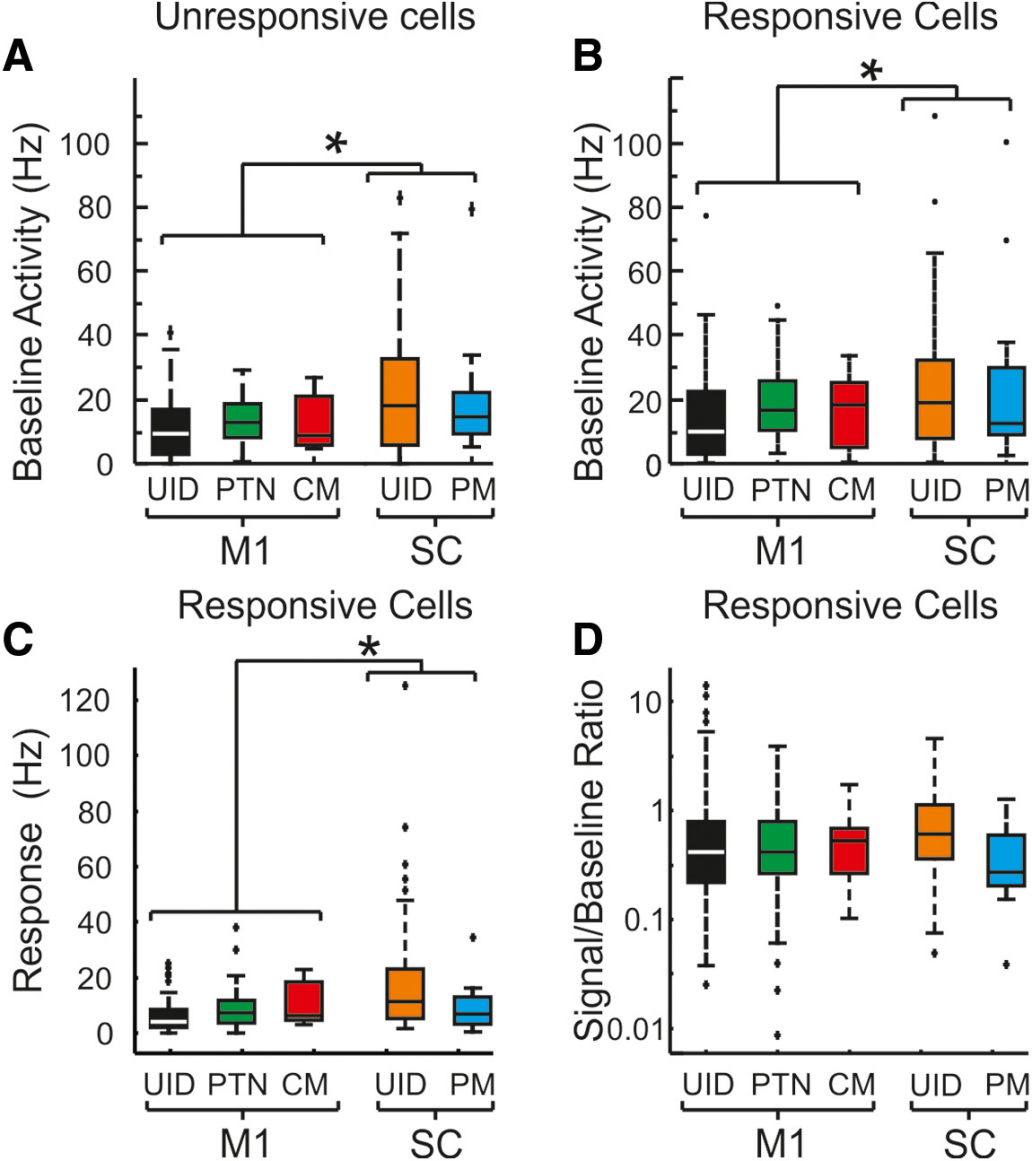
Baseline firing and response magnitudes in M1 and SC. ***A***, Boxplots of baseline firing rates for the different cell types indicated on the *x* axis. Only cells with no significant response to the perturbation are used. The same color code applies to remaining panels. ***B***, Same as in ***A***, but for cells that responded to the perturbation. ***C***, Absolute magnitude of the responses (relative to baseline, in spikes/s) for each cell type. For the purposes of this plot, the absolute value of response was used, such that cells with rate suppressions contribute positive values to the population. ***D***, Signal to background ratio (SBR), formed by dividing the response magnitude of (***C***) by the baseline rate in ***B*** for each cell type; note the logarithmic scale. UID cells in either M1 or SC, PTN. PM, Premotoneuronal cells in the SC. *p < 0.01.

Figure 9 compares the firing responses of cells between M1 and SC. Figure 9*A*, *B* shows boxplots of baseline firing rates for the different cell types (Fig. 9*A* is for cells without a significant response to the perturbation; and Fig. 9*B* is for responsive cells). Spinal neurons had significantly higher firing rates than cells in M1 (25 Hz vs 15.1 Hz, respectively, unpaired *t* test, *p* < 0.0001). A one-way ANOVA of baseline firing versus cell type for cells with a significant response to perturbation was significant (*F* = 4.05, *p* = 0.0035), and a *post hoc* analysis revealed that both spinal UID and PM cells had higher rates compared with M1 UID cells, but not PTNs or CM cells (Tukey-Kramer, adjusted for multiple comparisons, *p* < 0.05). A comparison of cells without any significant response to perturbations produced the same results (M1 vs SC, *t* test, *p* < 0.0001).

Figure 9*C* shows the magnitude of the responses (relative to baseline) for each cell type. For the purposes of this plot, the absolute value of response was used, such that cells with rate suppressions contribute positive values to the population. As with baseline firing, spinal neurons had a significantly higher response magnitude compared with M1 cells (10.2 Hz vs 5.2 Hz, respectively, unpaired *t* test, *p* < 0.0003). A one-way ANOVA of response magnitude versus cell type was significant (*F* = 9.1, *p* < 8 × 10^−7^). *Post hoc* analysis revealed that spinal UID cells had significantly higher response magnitudes compared with all other cell types (Tukey-Kramer, adjusted for multiple comparisons, *p* < 0.05).

To characterize how detectable a response to perturbation would be against baseline firing, we examined the ratio of the response relative to the background firing rate (signal to baseline ratio [SBR]) (Soteropoulos and Baker, 2007; Soteropoulos et al., 2012). This is useful, as the same response magnitude would clearly be easier to detect in a cell with low compared with a high firing rate. The values of SBR are shown in Figure 9*D*. There was no significant difference either between areas (all SC cells vs all M1 cells, SBR = 0.87 vs 0.89, respectively, unpaired *t* test, *p* > 0.1) or between different cell types (one-way ANOVA, *F* = 1.01, *p* = 0.4). This suggests that, relative to their background firing activity, cells in the SC responded to the perturbations just as robustly as cells in M1.

### Latency of responses to perturbation

**Figure 10.**
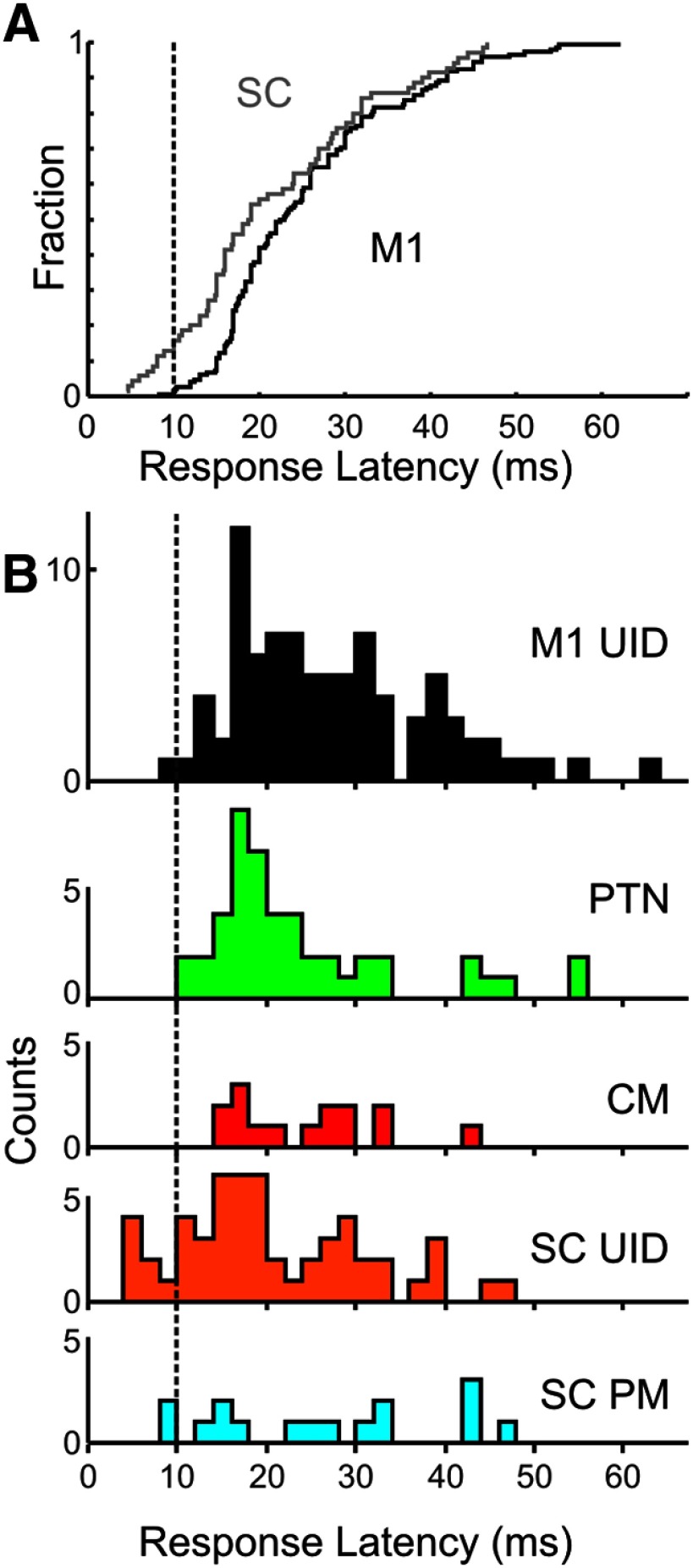
Response onset latency distribution. ***A***, Cumulative distribution of response latency following perturbation for SC cells (gray) and PTNs from M1 (black). The distributions were significantly different between areas (two-sample Kolmogorov-Smirnov test, *p* < 0.0013). ***B***, Distribution histogram of response latency for the different cell types. Dotted line indicates 10 ms latency for reference throughout.

The latency of the onset of neuronal responses to the perturbation is shown in Figure 10 for cortical and spinal cells. Figure 10*A* shows the cumulative distribution of response latency from all M1 (black line) and SC (gray) cells. There was significant difference between M1 and SC in terms of mean latency (unpaired *t* test, *p* < 0.017; mean latencies 21.7 ms vs 25.5 ms for SC and M1, respectively). There was substantial overlap in latencies between the cells from the two areas, although the SC had many more latencies < 10 ms than M1 (13% vs <1% of cells, respectively; Fig. 10*A*, left of dotted line).

The colored histograms in Figure 10*B* show the latency distributions for the different cell types (mean latency M1 UID, 27 ms; PTNs, 23.3 ms; CM cells, 24 ms; SC UID, 20.4 ms; PM cells, 26.3 ms). A one-way ANOVA comparing response latency with cell type was significant (*F* = 3.41, *p* = 0.009), and *post hoc* analysis revealed that the most significant difference was between the UID cells in M1 and the SC (Tukey-Kramer, adjusted for multiple comparisons, *p* < 0.05).

There was no significant difference in onset latency between SC cells that responded to cutaneous stimulation in the hand and those with responses to other types of stimulation or without sensory responses (one-way ANOVA, *F* = 0.67, *p* > 0.5).

### Contribution of spinal premotor and corticomotoneuronal cells to EMG responses

**Figure 11.**
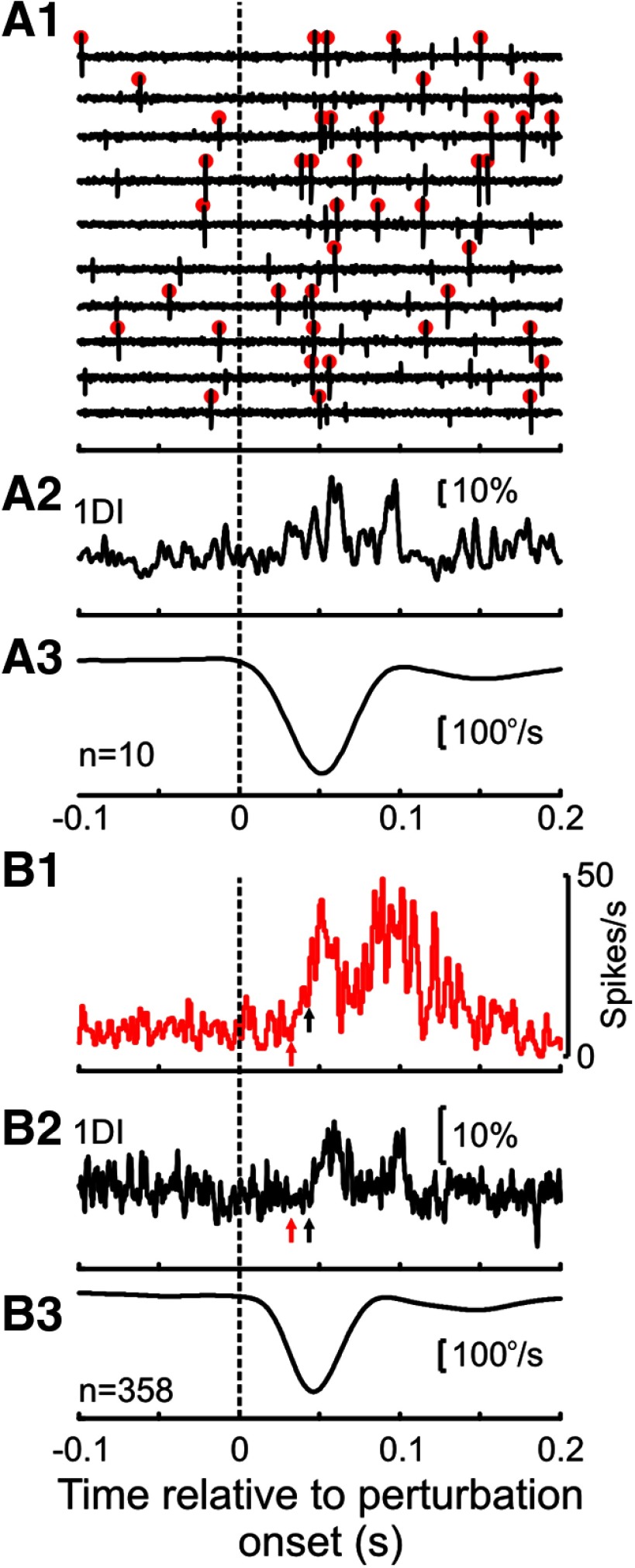
Exemplar spinal premotor neuron responding to index finger perturbation. ***A1***, Waveform traces from 10 mechanical perturbations. Red circles highlight the action potentials of this given cell. More than one cell was present in the recordings. ***A2***, Mean response of 1DI muscle (to which the cell was presynaptic to) for the same trials. EMG activity was normalized relative to preperturbation levels. ***A3***, Mean velocity trace of lever. ***B***, Same as in ***A***, but for all trials from which this cell was recorded. ***B1***, Mean response of cell shown in ***A***. ***B2***, mean response of 1DI muscle. ***B3***, mean velocity trace of lever. Red arrow indicates onset of neural response. Black arrow indicates onset of 1DI response. The time axis is the same for all subplots.

Because of concurrent recordings of EMG with neural activity, we were able to identify some cells as being presynaptic to motoneurons using spike-triggered averaging (see Materials and Methods). As some of these cells also responded to the perturbation, they could have contributed to muscle responses (Cheney and Fetz, 1984). An exemplar premotor spinal cell is shown in Figure 11. Figure 11*A* shows the raw recordings for 10 perturbations, and underneath is the mean EMG for those 10 perturbations from the muscle to which the cell was presynaptic; in this case, it was the 1DI muscle. Figure 11*B* shows the mean response of the same PM and muscle across all perturbations, indicating the earlier onset of the neural response compared with that of the muscle.

**Figure 12.**
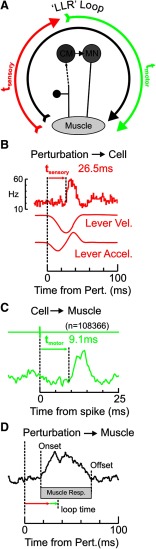
Estimation of sensorimotor loop time for a cell with direct linkage to motoneurons. ***A***, Simplified schematic demonstrating the “transcortical” loop for a CM cell following a sensory perturbation, through cell activation and back to muscle. A similar schematic would apply for PM cells. For simplicity, the afferent component is depicted as a single link, but the dotted line indicates that this is a polysynaptic path (for M1) or could be a monosynaptic or polysynaptic path (for SC). ***B***, Delay from perturbation to cell response for a CM cell in M1(t_sensory_). Top, The PETH for the given cell. Bottom, The mean velocity and acceleration traces. For this particular cell, the onset latency was 26.5 ms. ***C***, Delay from cell to muscle, estimated through spike-triggered averaging (t_motor_). Top, The trigger pulse from spike detection. Bottom, The average for the target muscle for this cell (1DI). In this example, the STA latency is 9.1 ms. ***D***, EMG response of target muscle to the perturbation, with onset and offset indicated by dotted lines. Gray bar represents the response epoch for this muscle. Red and green arrows indicate the estimated delays from ***B*** and ***C***, respectively. Their sum is the loop time (35.6 ms) for this particular neuron (indicated by the dotted line); this is the earliest time at which the response of this cell to the perturbation could start making a contribution to the response of the target muscle.

Although every action potential of CM and spinal PM cells generated a postsynaptic response in its target motoneurons, if the response of a cell to the perturbation occurred well after the EMG response, then a contribution would be far less likely than if the cell responded before or during the EMG response. In order to examine the cell response timing in this framework, we first estimated the reflex loop time for each cell (Fig. 12*A*). This was calculated to estimate the time it takes for the peripheral stimulus to reach the motoneurons, via the cell of interest.

To calculate the loop time for a given cell, we simply summed the delay with which the cell was activated following a perturbation (Fig. 12*B*, *t_sensory_*), with the delay estimated from spike-triggered averaging between the cell and its target muscle (Fig. 12*C*, *t_motor_*). For the example cell shown in Figure 11, these values are 26.5 ms and 9.1 ms respectively, giving a total of 35.6 ms total loop time. By measuring the offset and onset of the EMG response to the perturbation (Fig. 12*D*), the loop time could then be used to determine whether the cell could contribute to the EMG activation. For this example cell, the loop time was within the response period in the muscle, indicating that the cell could contribute to the EMG response.

**Figure 13.**
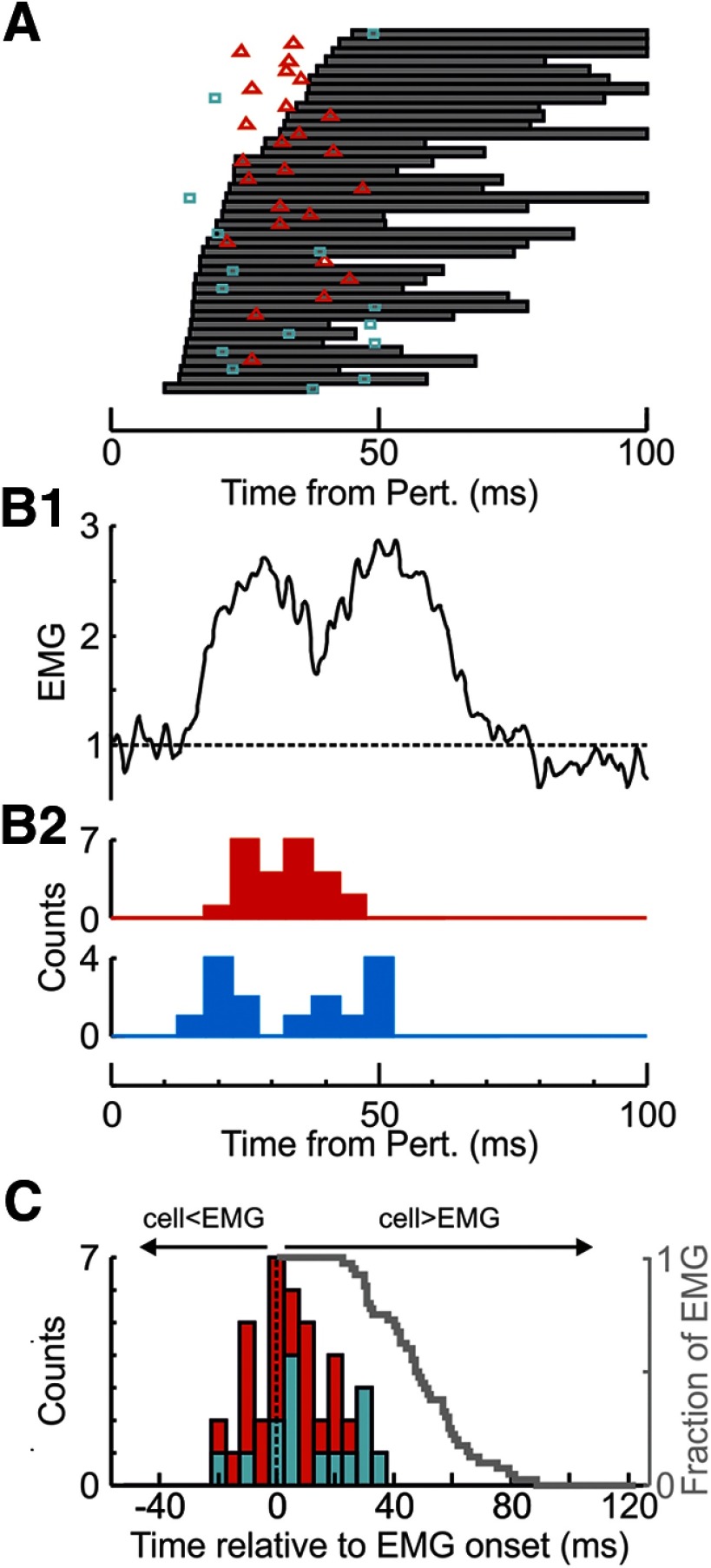
Population loop times for premotoneuronal cells. ***A***, Distribution of loop delays (sum of efferent delay and response delay to perturbation), compared with the onset and duration of muscle response to which cells were presynaptic. Red represents CM cells from M1. Cyan represents PM cells from the SC. Gray boxes represent the duration of the EMG response for the target muscle (truncated at 100 ms after perturbation). ***B1***, Mean EMG response across effects shown in ***A***. EMG was normalized as a fraction relative to preperturbation epoch. ***B2***, Histogram of loop times for CM (red) and PM (cyan) cells. ***C***, Histogram of loop times expressed relative to EMG response onset. Same color codes as in ***A***. Negative values indicate cell responses before EMG onset. The EMG responses were also aligned relative to their onset. Gray line indicates the fraction of effects responding at any given time after EMG response onset. As EMG responses had varying durations (as shown in ***A***), the fraction decreases with time.

Figure 13 shows the results of this analysis conducted for the CM/spinal PM cells that responded to the perturbation and whose target muscles also had clear responses. The same cell could contribute to more than one loop time estimate if it was presynaptic to multiple muscles, which in turn responded to the perturbation. Figure 13*A* shows the duration of the EMG response (gray squares) for the particular muscle that the PMs were presynaptic to and the colored markers show the loop delay. Red markers are for CM cells (*n* = 15), and cyan markers are for spinal PM cells (*n* = 11). Traces have been sorted in order of EMG onset latency.

All but two cells (both PM) have loop times before or during the EMG response period, consistent with a contribution to the EMG response. Figure 13*B1* shows the mean EMG response across all effects shown in Figure 13*A*. Figure 13*B2* shows the distribution of loop times.

The loop delay of all premotor effects was between 10 and 50 ms after the perturbation, and this was within 15 ms of the EMG onset for most effects (68%). In 30% of cases, the cell response began before the onset of EMG. Most EMG responses were long-lasting (range 22–86 ms), which is well beyond the width of most motor units recorded from the muscle surface by spike-triggered averaging from an intramuscular-recorded single unit (typically <25 ms) (Lemon et al., 1990; Baker and Lemon, 1998). This implies that the EMG response was composed of motor units firing at a range of delays. PMs whose contribution would reach the muscle after the onset of the EMG response could contribute to the activity of motor units activated later on in the response.

The data were replotted in Figure 13*C*, as a histogram of neural loop time relative to EMG onset latency. In addition, we also show the fraction of the cases where EMG activity continued at a given time after the onset. All EMG recordings showed continued activity for at least 22 ms after the response onset; by this time, a large fraction of responding PMs in both M1 (84%) and SC (66%) had loop times consistent with contributing to the response.

To summarize, these results show that the response onset of cells that are presynaptic to MNs, both in M1 and SC, occurs at delays that would allow most of the cells to contribute to the ongoing EMG response.

### Relative contributions of M1 and SC to EMG responses

Analysis from the previous section showed that PM responses from both M1 and SC occurred at latencies consistent with a contribution to the EMG responses. It is then of interest to examine the relative contribution of M1 and SC to the EMG response. It might be expected that SC cells would mostly contribute to the earlier components of the EMG response and M1 cells to the later components.

**Figure 14.**
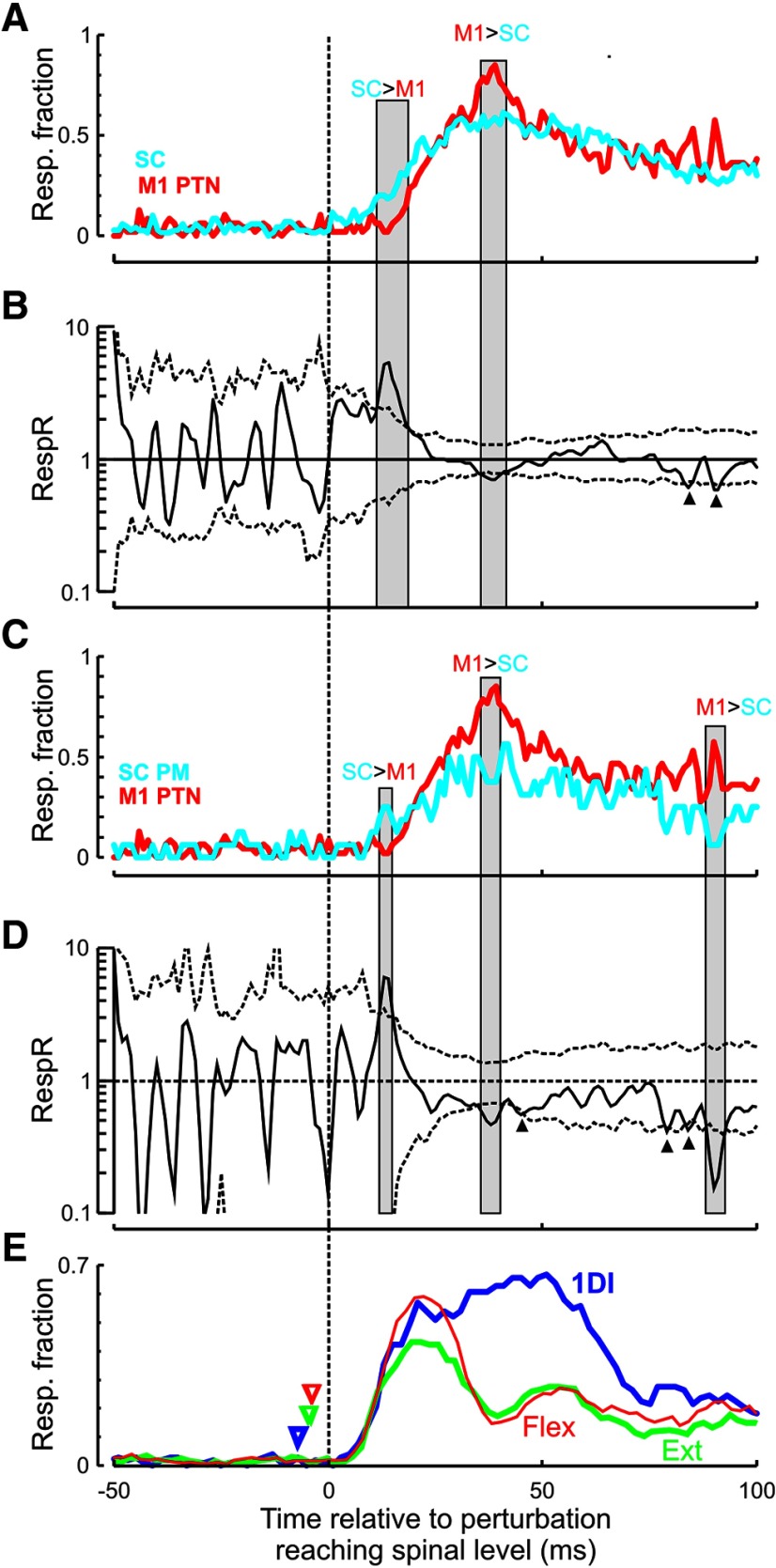
Relative responsiveness of M1 and SC. ***A***, Fraction of responsive cells in each area (red represents M1 PTNs: cyan represents SC) that are active after perturbation. ***B***, Ratio of the response fraction between the two areas (*RespR*; Eq. 1), plotted on a logarithmic scale. Dotted lines indicate the 95% confidence limits. PETHs were shifted according to their antidromic latencies before the curve for M1 was compiled. All times after perturbation are therefore for activity reaching the spinal level. There are two epochs of significant difference (11–21 and 35–41 ms; gray shading) with two additional brief crossings of the confidence limits (between 80 and 90 ms) highlighted by the small triangles. ***C***, Same as in ***A***, but only using spinal PM cells. ***D***, Same as in ***B***, showing three epochs of significant difference between areas (14–17, 35–41, and 88–93 ms). ***E***, Fraction of EMGs with significant activity. EMG response timings were shifted by subtracting the corresponding efferent delay for each muscle, thereby aligning EMG responses relative to the neural activity at the spinal level. Axis is thus the same as for ***C***, ***D***. Triangles represent the actual time of perturbation for the EMG traces. Muscle responses were grouped into 1DI (blue), muscles from extensor (Ext, green), and flexor (Flex, red) compartments in forearm.

To investigate the relative contributions of M1 and SC to responses, we compared the fraction of cells from each area that was significantly different from baseline at various delays after the perturbation onset, but to do so we need to take into account the added conduction delay from M1 to muscles. We could shift all M1 responses by ∼1.2 ms to account for this, as that would be the fastest delay from M1 to motoneurons in the cervical enlargement. However, our dataset included identified PTNs. As part of the identification process, we measured the antidromic latency following PT stimulation in the medulla, and subtracted 0.1 ms as utilization time. Since the pyramidal electrodes are approximately halfway between M1 and the cervical enlargement, this enabled us to calculate an accurate estimate for the delay from M1 to SC for each cell individually, simply by doubling the measured antidromic latency. The results of this analysis are shown inFigure 14*A*,*B*; the abscissa shows time relative to the perturbation, measured when responses reach the SC. Early after the perturbation (11-21 ms), the *RespR* was significantly >1 (mean 3.3), indicating that 3.3 times more SC cells than M1 cells contributed to the EMG response. Later on (35-41 ms), the *RespR* was significantly <1, suggesting that 1.3 times more M1 cells than SC cells contributed during this period. There were two further, brief, crossings of the confidence limits 80-90 ms after the perturbation (Fig. 14*B*, triangles); other than these epochs, there was no significant difference in the *RespR* during the rest of the 100 ms period after the perturbation.

In the same way that recordings in the cortex from UID cells are likely to be biased toward the larger pyramidal neurons, it is also possible that a similar bias exists in our spinal recordings from UID neurons. This might include ascending projection neurons, such as spinocerebellar cells, which would probably have larger extracellular spikes. To exclude this possibility, we repeated the analysis in Figure 14*A*, *B*, but we only used identified PMs from the SC compared with M1 PTNs; the results are shown in Figure 14*C*, *D*. As before, early after the perturbation (14–17 ms), premotor SC neurons have a significantly higher *RespR* value than M1 PTNs (mean of 4.7). A higher fraction of M1 PTNs is active later on (35–41 ms: mean 1.4, 88–93 ms: mean 1.9). Figure 14*E* shows, for comparison, the fraction of muscle recordings with activity significantly different from baseline. Results are shown by different lines for distal (1DI), forearm flexor (Flx), and extensor (Ext) muscles. The curves for each muscle have been adjusted by correcting by the respective efferent conduction delay; this means that all latencies are shown relative to activity in the SC, providing a consistent time frame for comparison with Figure 14*C*, *D*. The triangles show the actual time of perturbation for the different EMG traces.

To summarize, the very earliest part of the EMG response is dominated by the SC. There are brief (<7 ms) periods in the later part of the response with a slightly greater contribution of M1 than SC; but for most of the EMG response period, M1 and SC seem to make similar contributions.

### Do spinal cells respond directly to the perturbation, or indirectly to M1 activation?

Many SC interneurons are also known to receive descending inputs from the cortex (Lundberg and Voorhoeve, 1961; Jankowska et al., 1976; Riddle and Baker, 2010) and indeed PT terminations are densest in the intermediate layers of the SC in the monkey (Dum and Strick, 1996). It is thus possible that, for some spinal cells, the response after a perturbation is produced by descending activation from M1 instead of directly from afferent stimulation. We cannot directly address this, but we can exploit our knowledge of the antidromic latency of the PTNs to determine what fraction of SC cell responses were too early to have been initiated due to M1 activity. To do this, PTN responses to the perturbations were again adjusted by adding twice the antidromic latency to account for the conduction delay from the cortex to the SC. An additional 1 ms was added to account for the synaptic delay between corticospinal fibers and SC cells.

**Figure 15.**
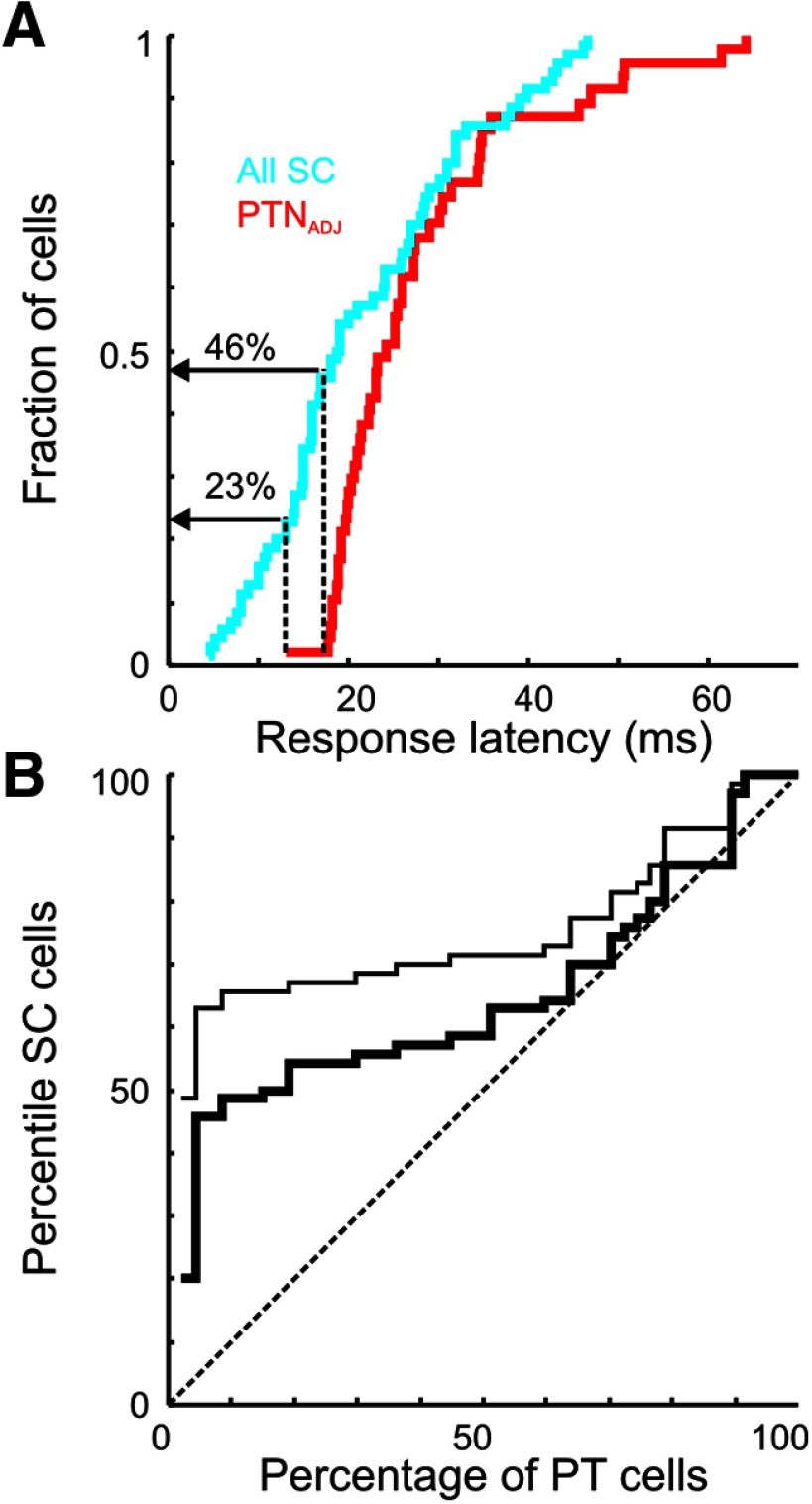
Comparison of response latency in M1 and SC. ***A***, Cumulative distribution of response latency following perturbation for all SC cells (cyan) and PTNs (red). PTN latencies were adjusted to include the conduction delay from cortex to SC based on the PTN-specific antidromic latency. The two distributions are significantly different (two-sample Kolmogorov-Smirnov test, *p* < 0.001). Vertical dotted lines indicate fraction of SC cells with latencies smaller than fastest and second-fastest PTN. ***B***, Percentile plot showing the percentage of SC cells with latencies smaller than a given percentage of PTN cells (thick black line). Thin black line indicates the percentage of SC cells with latencies smaller than a percentage of PTN cells, plus spinal cells from the superficial layers of the SC (depth < 0.5 mm), which are known not to receive any direct PT inputs.

The results of this analysis are shown in Figure 15. Figure 15*A* shows the cumulative distribution of SC (cyan) and adjusted PTN (red) response latency. The mean adjusted PTN response latency was 27.8 ms (range 13–64.2 ms). The two distributions were significantly different (two-sample Kolmogorov-Smirnov test, *p* < 0.001, KS statistic = 0.44); 23% of SC cells had onset latencies that were shorter than the earliest PTN-adjusted latency, which appeared to be an anomalous single measurement. Excluding that single PTN, 46% of SC cells had an earlier onset latency than the PTNs. This fraction is 38% if we only consider spinal PM cells. This relationship was examined further by a percentile plot (Fig. 15*B*). This shows the percentage of SC cells with latencies too early to be mediated by a given percentage of PTN cells (thick black line). This is clearly above the identify line(dotted), indicating that the SC cell population consistently responded earlier than PTNs.

In primates, there are relatively few corticospinal terminations from M1 in the most dorsal layers of the SC (Dum and Strick, 1996; Yoshino-Saito et al., 2010). If we assume that the most superficial SC cells (recorded <0.5 mm from the first cells encountered in the penetration) could not have responses mediated through M1, we can adjust the percentile plot accordingly. This amended plot is shown as in Figure 15*B* (thin line). The overall fraction of SC cells that could not have their responses initiated through any M1 PTN becomes >49% (rising to >63% if we exclude the earliest, potentially anomalous PTN response). These estimates should be considered conservative as they assume a monosynaptic connection; in reality, some effects from the corticospinal tract to SC cells will take a more indirect route, which would require a larger compensation than the 1 ms synaptic delay we allowed. Additionally, our dataset of PTN recordings is biased toward the fastest PTNs (Firmin et al., 2014; Innocenti et al., 2019; Kraskov et al., 2019a,b). If M1 contributes to SC responses through the many PTNs, which are even slower than those recorded here, then M1 contributions to SC responses would be even smaller than assessed here.

We conclude that a substantial fraction of SC responses to the perturbation were too early to be initiated by descending activation from PTNs. However, our results do not preclude a contribution to the latter components of the SC response, or to SC cells with late responses.

### Directionality Index

**Figure 16.**
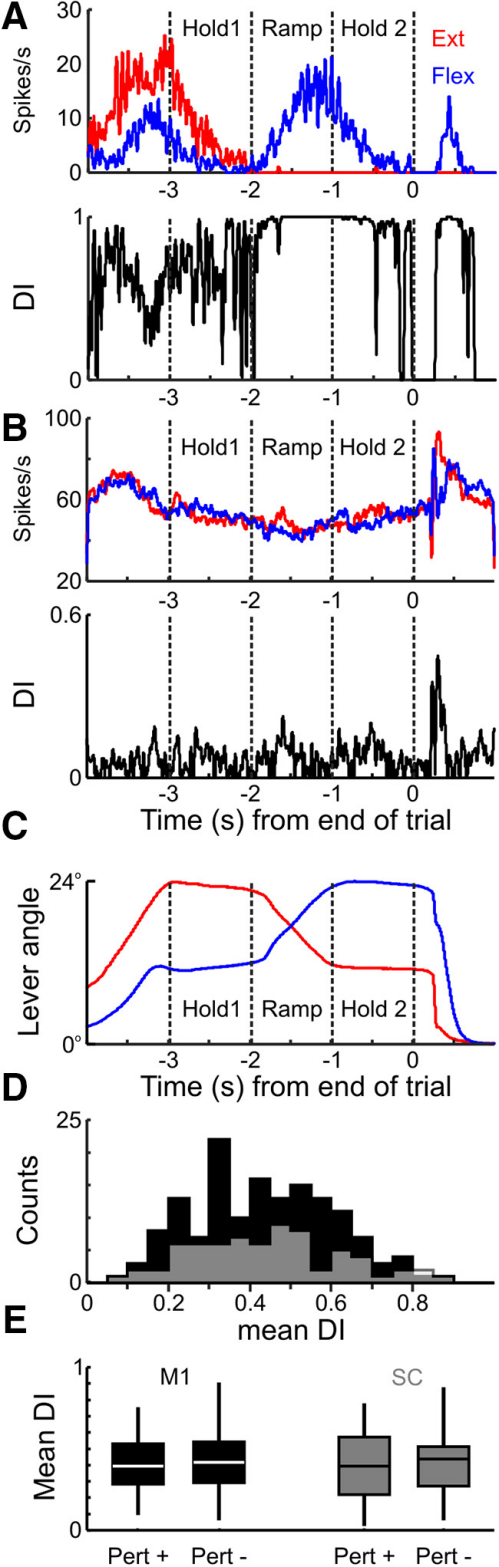
DI during task. ***A***, Top, PETH for example cell during flexion (blue) versus extension (red) trials. Bottom, The DI for the same cell; note the high DI values during the movement part of the task. ***B***, Same as in ***A***, but for a different cell with a lower DI as the cell responds very similarly during the two trial types. ***C***, Mean lever position signals for flexion (blue) and extension trials (red). The maximal lever angle corresponds to the finger being flexed. ***D***, Distribution of DI values for M1 (black) and SC cells (gray). ***E***, Boxplots showing the median DI (with 25th and 75th percentiles outlined by box) during the task for M1 (black) and SC cells (gray), divided into the population of cells that responded to the perturbation (Pert^+^) and those that did not (Pert^-^).

The results so far have shown that cells in both M1 and SC respond to mechanical perturbation; but if the cells that respond to the perturbation have minimal activity during the task, then that would limit the functional relevance of our results. To address this, we examined how active cells were during the task, and in particular whether cells were differentially active during flexion versus extensions trials by calculating a DI (see Materials and Methods). Cells that show a large modulation in firing during the task, but whose response was similar across flexion and extension trials, would result in a low DI value. These could be of lesser interest, as their activity could be postural and less directly related to task performance. The results of this analysis are shown in Figure 16.

Figure 16*A*, *B* (top) shows the PETH of two SC cells for flexion (blue) and extension (red) trials. Figure 16*A*, *B* (bottom) shows how the DI index modulates during the trial. The first cell (Fig. 16*A*) was very active during the ramp and second hold part of flexion trials, but inactive in these phases during extension trials. This resulted in a high DI (0.7). For the second cell (Fig. 15), although it had a very high firing rate overall and a clear response to the perturbation, the activity during the two movements was very similar (DI = 0.07). The vertical dotted lines in Figure 16*A*, *B* demarcate the movement phases of the task (Hold 1, Ramp, Hold 2), and Figure 15*C* shows the average lever angle signals for flexion (blue) and extension (red) trials.

Figure 16*D* shows the distribution of DI values for all M1 cells (black bars) and all SC cells (gray). There was no significant difference between the two areas (mean DI for M1: 0.42, for SC: 0.41, *p* > 0.1, unpaired *t* test). Most cells (>87%) in both areas had a DI value significantly different from zero, but there was no difference between cells with and without a response to the perturbation (Fig. 16*E*; one-way ANOVA, *F* = 0.44, *p* > 0.7). The same conclusions hold if we only consider spinal PM cells that do (mean DI = 0.45) and do not (mean DI = 0.44) respond to a perturbation (unpaired *t* test, *p* > 0.8). We conclude that most cells in our dataset were differentially active in flexion versus extension trials, including those that responded to the perturbation.

## Discussion

The neural origin of later components of the stretch reflex remains under debate. While a supraspinal contribution is well supported, our results affirm that the SC is also likely to play a role. SC cells, including premotor interneurons, responded as robustly as M1 neurons to a mechanical perturbation of the index finger, at latencies compatible with contributing to the EMG response. The response of SC neurons to mechanical perturbations has been rarely studied in the upper limb (Fetz et al., 2002); and to the best of our knowledge, this is the first time that SC neurons, including premotor interneurons, have been shown to respond to a mechanical perturbation in the fingers in the awake behaving primate. Below we consider some implications of this finding.

We should be cautious in assigning the spinal responses as arising from a single source. Many spinal neurons receive both afferent and descending inputs (Fig. 1), and these could both potentially contribute to spinal activity at different delays after the perturbation. For at least some of the spinal cells, it is highly likely that the initial response was mediated through sensory afferents as it was too rapid to be mediated through supraspinal pathways (Fig. 15). The rapid lever motion of our paradigm should be highly effective in activating Group Ia afferents, but other proprioceptive fibers (e.g., Group Ib and II) most likely also responded as during natural movements all three types can respond to rapid changes in muscle length (Dimitriou and Edin, 2008a,b), most likely due to fusimotor drive. Cutaneous afferents are also likely to contribute to some of the responses; indeed, most SC cells responding to cutaneous stimulation in the hand responded to the perturbation as well (Fig. 7*F*), and these cells were more superficially located, matching the known termination patterns of cutaneous afferents in the dorsal SC (Molander and Grant, 1985; Woolf and Fitzgerald, 1986; Edgley and Jankowska, 1988; Maxwell et al., 2000; Fisher et al., 2020).

A further contribution to the activation of spinal neurons could be made via descending pathways, originating from the cerebral cortex and brainstem (Lemon, 2008). These pathways project to motoneurons as well as to interneurons in the intermediate spinal laminae (Lundberg, 1979a; Alstermark and Isa, 2012). As these systems can also respond to perturbations (Soteropoulos et al., 2012; Herter et al., 2015), supraspinal corrective responses could thus reach motoneurons not just directly but also filtered through spinal interneurons. Descending contributions would require an extra delay for the sensory signal to reach the cortex or brainstem and the response to come back down to the SC. The SC therefore probably contributes to muscle responses for far longer than typically considered but should be best characterized as a mix of afferent and descending sources. Some human studies have also indicated a spinal contribution to the LLR (Lewis et al., 2004; Lourenco et al., 2006).

### Implications for motor control

A key feature of the LLR is its behavioral flexibility: the reflex amplitude can modulate according to the requirements of a manual task (Hammond, 1956; Pruszynski et al., 2008; Yang et al., 2011). Supraspinal areas are usually thought to subserve this because they not only respond to mechanical perturbations with permissive latencies, but are also highly active during task performance. In the cortex, M1 is well established to be a critical area for voluntary movements, particularly involving the distal forelimb, and many studies show that cells in M1 modulate their activity during action. Further down the neuraxis, the engagement of the red nucleus during voluntary reaching and grasping movements has been known for some time (Otero, 1976; Cheney, 1980; Gibson et al., 1985a,b; Alstermark and Isa, 2012), although the near-absence of a rubrospinal tract in humans (Nathan and Smith, 1982) makes rubrospinal contributions to the LLR unlikely in humans. The brainstem RF, once thought to contribute mostly to posture and locomotion, is now also known to be highly active during voluntary movements with the upper limb (Buford and Davidson, 2004; Schepens and Drew, 2004, 2006; Schepens et al., 2008), including during more isolated finger movements (Soteropoulos et al., 2012).

SC neurons are also active during voluntary movements. Cells in the SC can be highly active during nonmovement epochs in instructed delay tasks (Prut and Fetz, 1999), much like the premotor cortex (Kurata and Wise, 1988; Kalaska and Crammond, 1995; Crammond and Kalaska, 2000; Churchland et al., 2006) and RF (Buford and Davidson, 2004). Spinal interneurons are active during whole-arm movements, such as reaching to grasp (Riddle and Baker, 2010), as well as for more isolated wrist (Shalit et al., 2012) and grasping actions (Alstermark and Isa, 2012; Takei and Seki, 2010, 2013). Combined with the results of this study showing that SC cells respond to perturbations with permissive latencies, the SC thus fulfills the same criteria for contributions to the LLR as for M1.

Most experimental paradigms have examined upper limb LLR responses while subjects are seated and in a stable posture (but see Marsden et al., 1981). However, during everyday actions, unexpected perturbations need to be accommodated not just based on what the hands are doing but also on the involvement of the rest of the body, taking into account posture and any locomotion. It is well established that spinal circuits integrate inputs from multiple descending pathways, including systems that are critical for locomotion and posture, such as the reticulospinal and vestibulospinal projections (Illert et al., 1981; Krutki et al., 2003; Jankowska et al., 2005; Cabaj et al., 2006; Isa et al., 2006; Stecina et al., 2008; Bannatyne et al., 2009; Riddle and Baker, 2010) As such, SC premotor circuits are optimally placed to allow the LLR to be coordinated within a much broader behavioral context, although this remains to be explicitly shown.

Following motor damage, such as from stroke and SC injury, or in neurodegenerative conditions, such as Parkinson's disease, a substantial fraction of patients develop hypertonia and hyperreflexia (Angulo-Parker and Adkinson, 2018; McGregor and Nelson, 2019). Multiple systems along the neuraxis are likely to contribute to this, but our results lend further support to the evidence that changes in spinal circuitry could contribute to reflex gains (D'Amico et al., 2014).

### LLR: beyond a marker for cortical excitability?

The LLR is sometimes used as a noninvasive correlate of M1 excitability, although it has become clear over the last 20 years or so that its locus is far from singular (Pruszynski et al., 2011; Kurtzer, 2015). For distal and forearm muscles, contributions from M1 to the LLR are well supported, but there is also evidence for subcortical involvement (Soteropoulos et al., 2012). For more proximal muscles, there is also evidence for both M1 and subcortical contributions (Omrani et al., 2014; Herter et al., 2015; Foysal et al., 2016), but the relative importance of M1 is unclear. Although cells in M1 are modulated during proximal limb perturbations (Omrani et al., 2014; Pruszynski et al., 2014), in patients with aberrant bilateral corticospinal projections, bilateral LLRs are seen for distal (Matthews et al., 1990) but not proximal muscles (Fellows et al., 1996).

If all contributors to the LLR modulate in the same way during behavior, or if only M1 activity shows any task modulation, then there is little issue with using the LLR as a marker of M1 excitability. However, it is now well established that the state of both brainstem (Buford and Davidson, 2004; Soteropoulos et al., 2012) and the SC (Prut and Fetz, 1999; Takei and Seki, 2010, 2013; Williams et al., 2010) modulates with behavior. Although it remains to be shown whether the perturbation-evoked responses in subcortical regions modulate their size with behavioral context as they do in M1, particularly when subjects are comfortably seated in the laboratory environment, caution is warranted in assigning any observed task-related modulation in the LLR purely to cortical circuits.

In conclusion, our results show that a substantial fraction of neurons in the lower cervical SC respond to a mechanical perturbation of the index finger. The latency of these responses makes it highly likely that the SC contributes to the muscle responses beyond the SLR, at least for the forearm and intrinsic hand muscles examined here.
